# Ciprofloxacin for contacts of cases of meningococcal meningitis as an epidemic response: study protocol for a cluster-randomized trial

**DOI:** 10.1186/s13063-017-2028-y

**Published:** 2017-06-24

**Authors:** Matthew E. Coldiron, Gabriel Alcoba, Iza Ciglenecki, Matt Hitchings, Ali Djibo, Anne-Laure Page, Celine Langendorf, Rebecca F. Grais

**Affiliations:** 1 Epicentre, 8 rue Saint-Sabin, Paris, France; 2 Médecins Sans Frontières, 78 rue de Lausanne, Geneva, Switzerland; 3 Harvard T.H. Chan School of Public Health, Boston, MA USA; 4 Niamey National Hospital, Niamey, Niger

**Keywords:** Meningitis, meningococcal, Neisseria meningitidis, Ciprofloxacin, Epidemics, Drug resistance, bacterial, Niger

## Abstract

**Background:**

Epidemics of meningococcal meningitis are common in the “African meningitis belt.” Current response strategies include reactive vaccination campaigns, which are often organized too late to have maximal impact. A novel strain of *Neisseria meningitidis* serogroup C has been circulating in recent years, and vaccine supplies are limited. An evaluation of chemoprophylaxis with single-dose ciprofloxacin for household contacts of meningitis cases has therefore been recommended.

**Methods/design:**

A three-arm cluster-randomized trial has been designed for implementation during a meningococcal meningitis epidemic in a health district in Niger in which at least two Health Zones (HZs) have met the weekly epidemic threshold. The primary outcome is the incidence (attack rate) of meningitis during the epidemic. Villages will be randomized in a 1:1:1 ratio to one of three different arms: standard care, household-level prophylaxis, or village-wide prophylaxis. After study launch, when a case of meningococcal meningitis is identified in an HZ, the first reported case from a village will trigger the inclusion and randomization of the village. Household-level prophylaxis with single-dose ciprofloxacin will be offered in the home to all household members within 24 hours of the notification of the case, and village-wide distributions will occur within 72 hours of the notification of the case. The sample size necessary to detect differences between each of the two intervention arms and the standard care arm will be set after 4 weeks of data collection, in order to quantify multiple variables that could be particular to a given area. The primary analysis will compare attack rates at the end of the epidemic in each of the three arms. A nested sub-study will assess the effects of ciprofloxacin prophylaxis on the prevalence of ciprofloxacin-resistant enterobacteriaceae. A total of 200 participants in the standard care arm and 200 in the village-wide prophylaxis arm will provide stool samples at days 0, 7, and 28 following their village’s inclusion in the study.

**Discussion:**

An innovative trial is proposed for implementation during an epidemic that will assess the impact of a novel strategy for meningitis outbreak response. In parallel, we will describe potential negative effects of the intervention.

**Trial registration:**

ClinicalTrials.gov, NCT02724046. Registered on 15 March 2016. Last updated on 13 June 2017.

**Electronic supplementary material:**

The online version of this article (doi:10.1186/s13063-017-2028-y) contains supplementary material, which is available to authorized users.

## Background

### Meningococcal meningitis in sub-Saharan Africa and the emergence of serogroup C

Epidemics of meningococcal meningitis have been described in Africa since 1840 [1], and large-scale, cyclic epidemics have been described throughout what is called the African meningitis belt during the last century [2, 3]. Historically, the largest epidemics of meningococcal meningitis in the meningitis belt were caused by *Neisseria meningitidis* serogroup A (NmA). The introduction of a highly effective conjugate vaccine against NmA (MenAfriVac) in 2010 has had a major impact on the incidence of all-cause meningitis in the African meningitis belt [4], dramatic effects on the incidence of NmA [5], and has virtually eliminated its nasopharyngeal carriage [6].

In the relative calm after the introduction of MenAfriVac, small-scale epidemics caused by a novel strain of *N. meningitidis* serogroup C (NmC) were seen in northwest Nigeria in 2013 and 2014 [7]. These two outbreaks were followed by a much larger epidemic in 2015 in both Nigeria (>6000 cases reported) and Niger (>9000 cases reported) [8].

The response to the 2015 epidemic was complicated by the scarcity of vaccines against NmC. This limited availability may continue for several years, during which time the risk of repeated NmC epidemics is expected to be high [9].

### Prevention of meningococcal meningitis

Two main strategies exist for meningitis prevention: mass vaccination (either in a preventive manner such as the roll-out of MenAfriVac, or in reactive campaigns during epidemics) and antibiotic prophylaxis. Recommendations for the use of these two strategies vary greatly depending on context.

In the African meningitis belt, the emphasis has historically been on reactive mass vaccination campaigns. Unfortunately, because these campaigns can be difficult to organize, and because epidemics due to NmA were often explosive and short-lived, the impact of such campaigns has been called into question [10, 11]. Shortening the delay between epidemic declaration and vaccination could potentially have an important impact [12], but logistical and operational hurdles are difficult to overcome in many contexts.

On the other hand, antibiotic chemoprophylaxis was implemented in the pre-vaccine era in sub-Saharan Africa [1] and is commonly used among household contacts of cases during outbreaks in western countries [10, 13], but it is not recommended as part of the usual response to meningitis outbreaks in sub-Saharan Africa [14]. This recommendation has been made based on expert opinion that implementing prophylaxis would divert efforts from case management and reactive vaccination campaigns. Nonetheless, data from the 2015 epidemic in Niger suggested that attack rates for household contacts were approximately 20 times higher than that of the general community, suggesting that this group could be an important target for prevention activities [15]. On the other hand, in the same epidemic, 86% of households reported only one case, which could mean that the public health impact of targeting only household contacts could be limited. A recent meta-analysis of four small studies showed a substantial reduction in subsequent cases of meningitis in households receiving chemoprophylaxis [16]. Three antibiotic regimens have been considered as a prophylaxis in other settings: single-dose oral ciprofloxacin, single-dose injectable ceftriaxone, and a 2-day, four-dose course of oral rifampicin. The logistical problems inherent with using an injectable drug like ceftriaxone on a large scale and the unavailability of rifampicin as a single drug in countries with a high prevalence of tuberculosis make ciprofloxacin the best choice for potential prophylactic use in meningococcal meningitis outbreaks in Africa.

### Study rationale

Because of the threat from NmC and the relative scarcity of NmC-containing vaccine for the foreseeable future, an expert group convened by the World Health Organization (WHO) called for a clinical trial to document the effectiveness of the use of ciprofloxacin chemoprophylaxis as an epidemic response in sub-Saharan Africa [9]. Given that one concern about the widespread use of ciprofloxacin would be the development of fluoroquinolone-resistant bacteria (particularly in the gastrointestinal tract), we will carry out a parallel sub-study to assess the community-wide prevalence of ciprofloxacin-resistant enterobacteriaceae over time.

We have designed a cluster-randomized trial in response to this call. The proposed trial surpasses the original recommendation of household-level chemoprophylaxis by adding an additional evaluation of village-level chemoprophylaxis, which may be easier to implement and more effective in an epidemic context. Cluster-randomized trials of preventive interventions for both infectious and non-infectious diseases have been implemented in many different African settings [17–20]. We have also designed a sub-study to respond to one of the caveats to this call for a trial, which will investigate the effect of ciprofloxacin chemoprophylaxis on the selection of antimicrobial resistance.

## Methods/design

### Trial aims and objectives

The study is designed as a cluster-randomized trial with three parallel arms in the setting of a future meningitis epidemic. The primary objective is to compare the incidence of meningitis among villages receiving standard care, household single-dose ciprofloxacin prophylaxis, and village-wide single-dose ciprofloxacin prophylaxis. Secondary objectives include comparing the incidence of meningitis between villages in the three arms by sex and age.

**Fig. 1:**
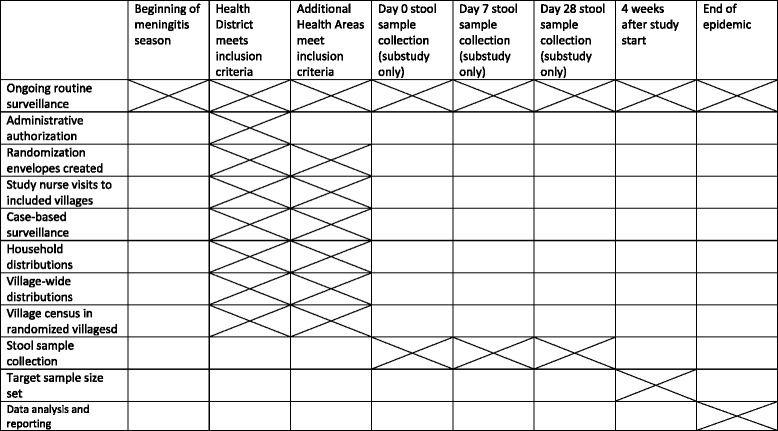
SPIRIT figure

A schedule of trial interventions, the Standard Protocol Items: Recommendations for Interventional Trials (SPIRIT) figure, is presented in Fig. 1, and a populated SPIRIT checklist is available in Additional file 1.

### Study setting and launch criteria

The study will be implemented in a Health District (HD) experiencing a meningitis outbreak. In order to launch the study protocol, at least two Health Zones (HZs) of the HD will have met the weekly meningitis epidemic threshold [14]. All villages in HZs included in the study will be eligible for inclusion. A village will be randomized to a study arm and receive its intervention once the first case has been reported from that village after the study has opened in an HZ. On inclusion in the study, a dedicated study nurse and at least two dedicated community health agents who have been trained in study procedures and case definitions will be stationed in each HZ.

### Selection of villages and randomization procedures

Because it is impossible to predict which villages will notify cases in advance of an epidemic, the randomization list will not be prepared in advance, so as to ensure a balanced randomization. Villages will be randomized in a 1:1:1 ratio to receive standard care, household prophylaxis or community-wide prophylaxis.

Once an HZ has met inclusion criteria, a set of sequentially numbered, sealed envelopes will be prepared, each containing the name of an intervention arm. When the first case presents from a village, study staff in the Health Center (HC) will telephone a central randomizer, who will be otherwise independent of the study functioning.

The central randomizer will keep a master list of names of villages in each HZ and their study assignments. A reference list of village names and study assignments will be updated in each HC as villages are included. When a case presents, study staff based in the HC will verify whether the case-patient’s village of origin has already been randomized. This information will in turn be double-checked against the central-level randomization list.

### Study interventions

In the standard care arm, after the notification of the first case from a village, a study nurse will visit the village and lead an informational session focusing on the signs and symptoms of meningitis, as well as the urgency of presenting to the nearest HC if any of those symptoms occur.

In the household-level prophylaxis arm, each time a case is notified, a study nurse will visit the case’s household within 24 hours. A household is defined as a group of people living in the same building or group of adjacent buildings and under the authority of a single head of household. In the case of polygamy, all wives (and their children) of a male head of household will be considered to be members of a single household, provided that they live in the same building or group of adjacent buildings. In the case of multiple heads of household living in one compound of houses, only persons living under the authority of the head of household of the declared meningitis case will be considered a household member and therefore invited to take ciprofloxacin. The nurse will offer ciprofloxacin to all persons currently living in the household and present at the time of the visit, and supervise intake if they accept. (Household members who have accompanied the notified patient will be offered ciprofloxacin at the HC.) Return visits will be arranged for absent household members. In villages randomized to this arm, prophylaxis of a reported case-patient’s household will occur with each subsequent case notified from that village.

In the community-wide prophylaxis arm, after notification of the first case, a study nurse will visit the village within 24 hours and meet with community leaders. During this visit, a community-wide distribution of ciprofloxacin will be arranged within 72 hours of the initial case presentation at the HC. These distributions will be organized with local authorities, and they will take place after a series of community meetings explaining the study and the distribution to villagers. In this arm, distribution of ciprofloxacin will occur only once, after the first notified case-patient in a village.

### Ciprofloxacin and its administration

A single dose of oral ciprofloxacin will be administered using age-based dosing (see Table 1). All doses will be directly observed. Tablets will be administered with water. Children unable to swallow pills will have the tablet crushed and dissolved in sweetened water.

**Table 1:** Age-based dosing of ciprofloxacin

Age	Dose (mg)	Formulation
≥12 years	500	1 tablet
5–11 years	250	1 tablet
1–4 years	125	½ tablet (250 mg tablet)
3–11 months	100	2 ml oral suspension (250 mg/5 ml)
<3 months	75	1.5 ml oral suspension (250 mg/5 ml)

### Standard care for meningitis

Throughout the study period, the diagnosis and treatment of meningitis (including referral) will be provided free of cost throughout the study area. Diagnosis and treatment will follow national protocols. Participation in the study will not affect the eligibility of a village or an HZ for reactive vaccination.

### Meningitis surveillance

Once an HZ has been included in the study, a case-based surveillance system will be put in place in HCs, or reinforced if one is currently functional. Dedicated study staff will be placed in each facility to carry out this system.

The following case definitions will be used:

- Suspected case: Abrupt fever (>38.5 °C rectal or >38.0 °C axillary) and at least one of the following signs: neck stiffness, floppy neck, bulging fontanelle, convulsions, or other meningeal signs
- Confirmed case: Isolation or identification of a causative organism (*Neisseria meningitidis, Streptococcus pneumoniae, Haemophilus influenzae* serotype B) in the cerebrospinal fluid (CSF) of a suspect case by culture, polymerase chain reaction (PCR), or agglutination test

Demographic and clinical information will be collected for each suspected case, and a unique identifier will be assigned following national protocols. The village of residence will be directly confirmed with the patient or their caregivers. Information about receipt of ciprofloxacin will also be collected.

Lumbar puncture and CSF analysis will occur following standard protocols and procedures. Any information collected as a result of this procedure will enter into the national surveillance system and will be crossed with local databases. In addition, in order to obtain accurate denominators for the calculation of attack rates, a team of community health workers will perform a census in each village included in the study.

### Sample size

Meningitis incidence over the course of the epidemic will be expressed as an attack rate (proportion of the population developing meningitis over the defined time period). The sample size needed to show a difference in the reduction of the meningitis attack rate depends on multiple factors, some of which can be reliably estimated and others which cannot be reliably estimated in advance (distribution of village sizes in the study zone, overall amplitude of epidemic) [21].

Additional file 2 presents simulations of the number of clusters necessary given specified attack rates and impact of the ciprofloxacin intervention, assuming an alpha (α) error of 5%, 90% power, and an inter-cluster correlation coefficient of 0.025 [22, 23]. The most likely parameters would need less than 75 villages per arm, which is historically a reasonable number of villages affected in meningitis outbreaks in HDs in Niger and in neighboring countries in the meningitis belt.

A provisional target sample size will be set, but because of these multiple uncertainties, the final target sample size will be set after four weeks of accumulated study data. This period would be sufficiently long to allow for a description of the demographic co-factors in the study area. Additional file 2 shows high variability depending on these co-factors, but the most likely scenarios present sample sizes that are logistically feasible.

### Resistance sub-study

A total of 200 participants in the standard care arm and 200 participants in the community-level prophylaxis arm will be enrolled in the resistance sub-study. Three stool samples will be collected from each participant, at days 0, 7, and 28 using standard operating procedures (SOPs) developed for stool sample collection in Niger. Samples from day 0 in the community-level prophylaxis arm will be collected prior to distribution of ciprofloxacin.

Assuming baseline prevalence of ciprofloxacin-resistant enterobacteriaceae of 20%, a sample size of 131 persons in each of the two arms would have 90% power to detect a change in the prevalence to 30% over the three repeated measures. Assuming an attrition rate of 25% and 20% of improperly collected samples, a sample size of 200 persons per arm would allow for the calculation of the primary resistance objective (community-wide prevalence of ciprofloxacin-resistant enterobacteriaceae) with sufficient power.

Twenty participants in each of 20 villages (10 villages in the standard care arm, 10 villages in the community-wide prophylaxis arm) will be included. In each of the arms, the resistance sub-study will begin once at least 10 villages have notified cases.

Once the resistance sub-study has begun, the first odd-numbered village which notifies a case each day in each of the two arms will be included in the sub-study. An exhaustive list of households will be prepared in these villages, and 20 households will be randomly selected from this list. One member of each household will be randomly selected and will be invited to participate in the resistance sub-study. Only participants who know in advance that they will be absent during follow-up visits will be excluded. If this is the case, a second member of the household will be randomly selected as a replacement.

A stool sample container will be distributed to each participant the day before sample collection, together with instructions for collecting the sample the following morning. Study staff will then return to the village to collect the fresh stool samples. The same procedures will be followed on days 7 and 28. In villages receiving ciprofloxacin prophylaxis, day 0 samples will be collected up to the time of ciprofloxacin administration in the village. At days 7 and 28, samples will also be accepted the day following the scheduled sample collection (i.e., days 8 and 29), if the participant is not able to provide a sample on the scheduled day.

### Sample processing

The processing of stool samples will depend on the location of the study and will be detailed in the study SOPs. In brief, stool samples will be collected by study staff and either stored at 4 °C or inoculated in a transport medium until reception in the central laboratory. Stool samples will then be plated on a MacConkey agar plate containing ciprofloxacin. After incubation, colonies will be identified using standard microbiological methods. The minimum inhibitory concentration of ciprofloxacin and third-generation cephalosporins will be determined [24], and 10% of samples will be sent to a reference laboratory for quality control purposes.

### Statistical analysis

The primary analysis, pairwise comparisons of attack rates, will be made between each intervention group using the Student’s *t* test, weighted to account for varying cluster size, and following an intention-to-treat basis. If the assumption of normality does not hold, we will consider a transformation of the data or a non-parametric test such as the Wilcoxon rank-sum test. Attack rates in each arm will be expressed as a weighted mean of cluster-specific attack rates, and will be presented with their 95% confidence intervals. Each of the two intervention groups will be independently compared to the standard care arm. The overall effect of prophylaxis in the intervention arms will be calculated as OEP = (AR_0_ – AR_1_)/AR_0_, where AR_0_ and AR_1_ are the weighted average attack rates in the control and treatment arm respectively [25]. The direct effect of ciprofloxacin prophylaxis will be estimated by comparing attack rates among persons receiving ciprofloxacin versus those not receiving ciprofloxacin in the household-prophylaxis arm.

The study will take place during an epidemic, and events beyond the control of the study sponsor could affect the propagation of the meningitis epidemic. If major events that could affect the course of the epidemic occur (early rains, a vaccination campaign, etc.), a binary variable (before/after the event) will be added to the data and controlled for using a cluster-level regression model.

The analysis of the resistance sub-study will be done on the basis of the per-protocol population for whom all three stool samples are available. The primary sub-study outcome (change in community-wide prevalence of carriers of ciprofloxacin-resistant enterobacteriaceae) will be compared using the method described by Liu and Wu [26]. Individual-level acquisition rates at 7 and 28 days following the initial sample will also be calculated with corresponding 95% confidence intervals using the binomial distribution. Acquisition will be defined as the presence of ciprofloxacin-resistant enterobacteriaceae at day 7 or day 28 in a participant who did not have ciprofloxacin-resistant enterobacteriaceae at the time of baseline sample collection. A Fisher exact test will be used to compare proportion of acquisition between study interventions. All tests will be two-sided with a significance threshold of α = 0.05.Due to the uncertainty of several parameters used in the sample size calculation, we plan to recruit for four weeks and perform a sample size re-estimation based on the estimated value of *p*_0_, the attack rate in the controls, from this sub-sample. For this, we follow the method of Lake et al. [27]. We make two assumptions in doing this: that epidemics in individual villages last only a short time, meaning the *p*_0_ estimated 4–6 weeks after recruitment of a village will be very close to *p*_0_ for that village at the end of the study; and that the attack rate in each village will remain constant during the whole course of the epidemic, i.e., that villages affected early in the epidemic do not have systematically bigger or smaller epidemics than villages affected later in the epidemic.

### Safety reporting

Passive surveillance for serious adverse events (SAEs) will be conducted from the start of the study through 28 days following the last dose of ciprofloxacin administered, and be carried out by study staff in HCs.

An SAE is defined as any untoward medical occurrence that, at any dose of ciprofloxacin received, results in death, is life threatening, requires inpatient hospitalization or prolongation of existing hospitalization, or results in persistent or significant disability or incapacity, or any medically important event/reaction that may jeopardize the subject and may require medical or surgical intervention to prevent one of the outcomes listed above.

### Informed consent and other ethical considerations

Procedures for obtaining informed consent have been developed in accordance with the Ottawa Statement on the Ethical Design and Conduct of Cluster Randomized Trials [28]. Gatekeepers have been defined as the health care professional in charge of the HC and village chiefs and their deputies. Village chiefs will be asked to provide written permission for the randomization of their village. It will not be possible to obtain individual-level informed consent for participation, as each arm will likely include several thousand individuals during an emergency. We have met established criteria for the waiver of individual consent in a cluster-randomized trial [28, 29]. Nonetheless, in villages receiving household chemoprophylaxis, the study nurse will present the risks and benefits of taking ciprofloxacin to household members, and it will be administered only to those who are willing. In the village-wide prophylaxis arm, a series of community meetings will be held in advance of the mass distribution, during which the study and its risks and benefits will be presented and questions may be answered. Those persons who present to the ciprofloxacin distribution site will be considered to have been duly informed of the risks and benefits of taking ciprofloxacin and the voluntary nature of their participation.

### Trial organization and administration

The trial was developed by Epicentre with the support of Médecins Sans Frontières. The primary trial sponsor is Epicentre, who will hold the data and conduct all analyses. The primary sponsor will independently monitor study execution at field sites. A scientific committee has been convened to review drafts of the protocol and analytic plan, and will be consulted for queries regarding implementation and analysis during and after the trial.

When the trial report is completed, the investigators will share the summary results with local, regional, and national health authorities. The findings from this study will also be published in a peer-reviewed scientific journal and disseminated at appropriate conferences. The ultimate decision to submit a manuscript will remain with the primary sponsor.

The research data will be the property of the sponsors, but data will be made as widely and freely available as possible, while safeguarding the privacy of participants and protecting confidential data. A de-identified dataset can be made available under a data-sharing agreement that provides for a commitment to using the data only for research purposes and securing data using appropriate technology.

## Discussion

Despite the undeniable success of MenAfriVac, the emergence of NmC as a potential cause of major epidemics in the African meningitis belt has brought new problems. Limited supplies of vaccine against NmC, combined with limitations inherent to organizing mass vaccinations (particularly regarding their timing), have led to a recommendation to assess the use of chemoprophylaxis among household members as an alternative epidemic response.

The proposed trial responds to this call, and goes one step further by adding a third arm in which chemoprophylaxis would happen at a village level following the notification of the village’s first case. This addition responds to on-the-field realities in many parts of the meningitis belt, where it may be politically difficult to treat only one household in a small village when other villagers would also be at risk. Also, despite the higher risk for household members, most households will still report only one case during an epidemic, so this community-wide strategy may have a greater impact on overall meningitis incidence.

On the other hand, given the growing problem of antibiotic resistance, even if this trial were to show overwhelmingly positive results, it would be impossible to recommend chemoprophylaxis as a routine epidemic response without having evidence on the possible effect of mass distributions of ciprofloxacin on the prevalence of resistant pathogens. We believe that by carrying out the sub-study during the epidemic we will provide the most realistic evidence on which to base potential decision making.

The evidence gained in this trial will provide important information about a subject that is very poorly described in the literature, and while we hope that it would never need to be implemented, we are ready to do so.

### Trial status

All resources, materials, and authorizations necessary for the trial are in place in Niger, and surveillance is ongoing to detect eligible districts during upcoming meningitis epidemics. We are currently considering submitting the protocol in other meningitis belt countries.

## Additional files

Additional file 1: Populated SPIRIT checklist. These are our responses to the standard SPIRIT checklist. (DOC 121 kb)

Additional file 2: Sample sizes necessary for the trial given different conditions. This table presents a range of different possibilities for sample size necessary for the trial, depending on various conditions, some of which will not be known until after the trial’s start. (XLSX 22 kb)

## Abbreviations

95% CI: 95% confidence interval
HC: Health Center
HD: Health District
HZ: Health Zone
NmA: *Neisseria meningitidis* serogroup A
NmC: *Neisseria meningitidis* serogroup C
PCR: Polymerase chain reaction
